# Supplementary material for: Echinochrome Prevents Sulfide Catabolism-Associated Chronic Heart Failure after Myocardial Infarction in Mice
Source: Mar Drugs. 2023 Jan 12;21(1):52. doi: 10.3390/md21010052 (PMC9863521; doi:10.3390/md21010052)
Supplement: Supplementary file 1 [file marinedrugs-21-00052-s001.zip › marinedrugs-2030823-supplementary.pdf]

Supplementary Materials

Echinochrome prevents sulfide catabolism-associated chronic heart failure after myocardial infarction in mice

Xiaokang Tang, Akiyuki Nishimura, Kohei Ariyoshi, Kazuhiro Nishiyama, Yuri Kato, Elena A Vasileva, Natalia P. Mishchenko, Sergey A. Fedoreyev, Valentin A. Stonik, Hyoung-Kyu Kim, Jin Han, Yasunari Kanda, Keitaro Umezawa, Yasuteru Urano, Takaaki Akaike and Motohiro Nishida

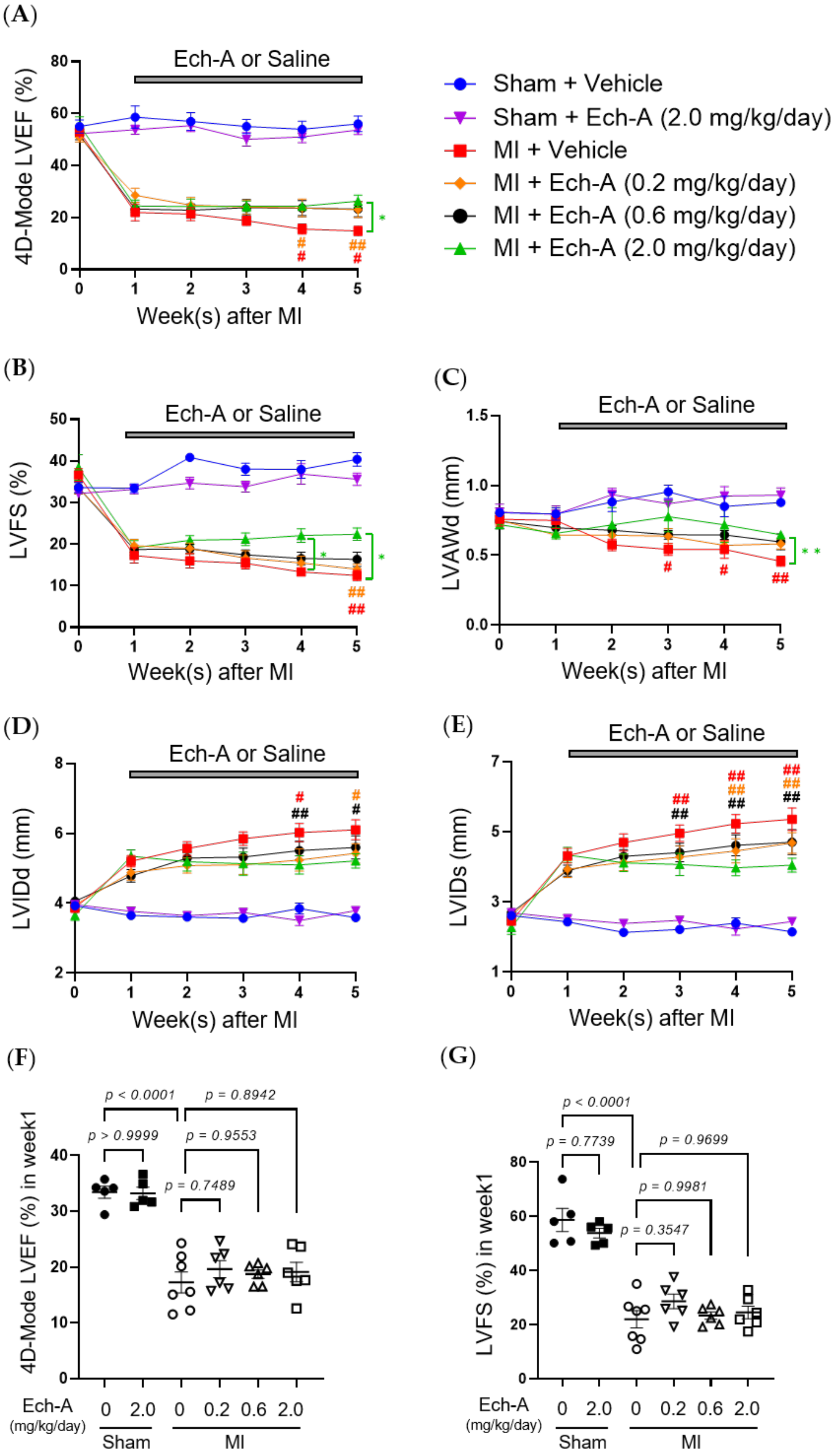
(A)
Ech-A or Saline
4D-Mode LVEF (%)
Week(s) after MI
Sham + Vehicle
Sham + Ech-A (2.0 mg/kg/day)
MI + Vehicle
MI + Ech-A (0.2 mg/kg/day)
MI + Ech-A (0.6 mg/kg/day)
MI + Ech-A (2.0 mg/kg/day)
(B)
Ech-A or Saline
LVFS (%)
Week(s) after MI
(C)
Ech-A or Saline
LVAWd (mm)
Week(s) after MI
(D)
Ech-A or Saline
LVIDd (mm)
Week(s) after MI
(E)
Ech-A or Saline
LVIDs (mm)
Week(s) after MI
(F)
4D-Mode LVEF (%) in week1
p < 0.0001
p = 0.8942
p > 0.9999
p = 0.9553
p = 0.7489
Ech-A
(mg/kg/day)
0 2.0 0 0.2 0.6 2.0
Sham
MI
(G)
LVFS (%) in week1
p < 0.0001
p = 0.7739
p = 0.9699
p = 0.9981
p = 0.3547
Ech-A
(mg/kg/day)
0 2.0 0 0.2 0.6 2.0
Sham
MI

Figure S1. Temporal changes in echocardiographic parameters. (**A–E**) Changes in 4D-mode LV ejection fraction (LVEF) (**A**), LV fractional shortening (LVFS) (**B**), LV anterior wall thickness at end diastole (LVAWd) (**C**), LV internal diameter at end diastole (LVIDd) (**D**) and end systole (LVIDs) (**E**) in mice after MI. Osmotic pump filled with Ech-A or vehicle was implanted intraperitoneally 7 days after MI. (**F,G**) Quantification of LVEF (**F**) and LVFS (**G**) at 1 week after MI (before Ech-A administration). (Sham + vehicle (●): n=5, Sham + Ech-A 2.0 mg/kg/day (■): n=5, MI + vehicle (○): n=7, MI + Ech-A 0.2 mg/kg/day (▽): n=6, MI + Ech-A 0.6 mg/kg/day (△): n=6, MI + Ech-A 2.0 mg/kg/day (□): n=6). Data were presented as mean ± s.e.m. *$P<0.05$, **$P<0.01$ using two-way ANOVA followed by Šídák's multiple comparison test, compared to the value in the MI-Vehicle group (**A–E**). #$P<0.05$, ##$P<0.01$ using two-way ANOVA followed by Dunnett's multiple comparison test, compared to the respective value at 1 week (**A–E**). P-values were calculated using one-way ANOVA followed by Šídák's multiple comparisons test (**F,G**).

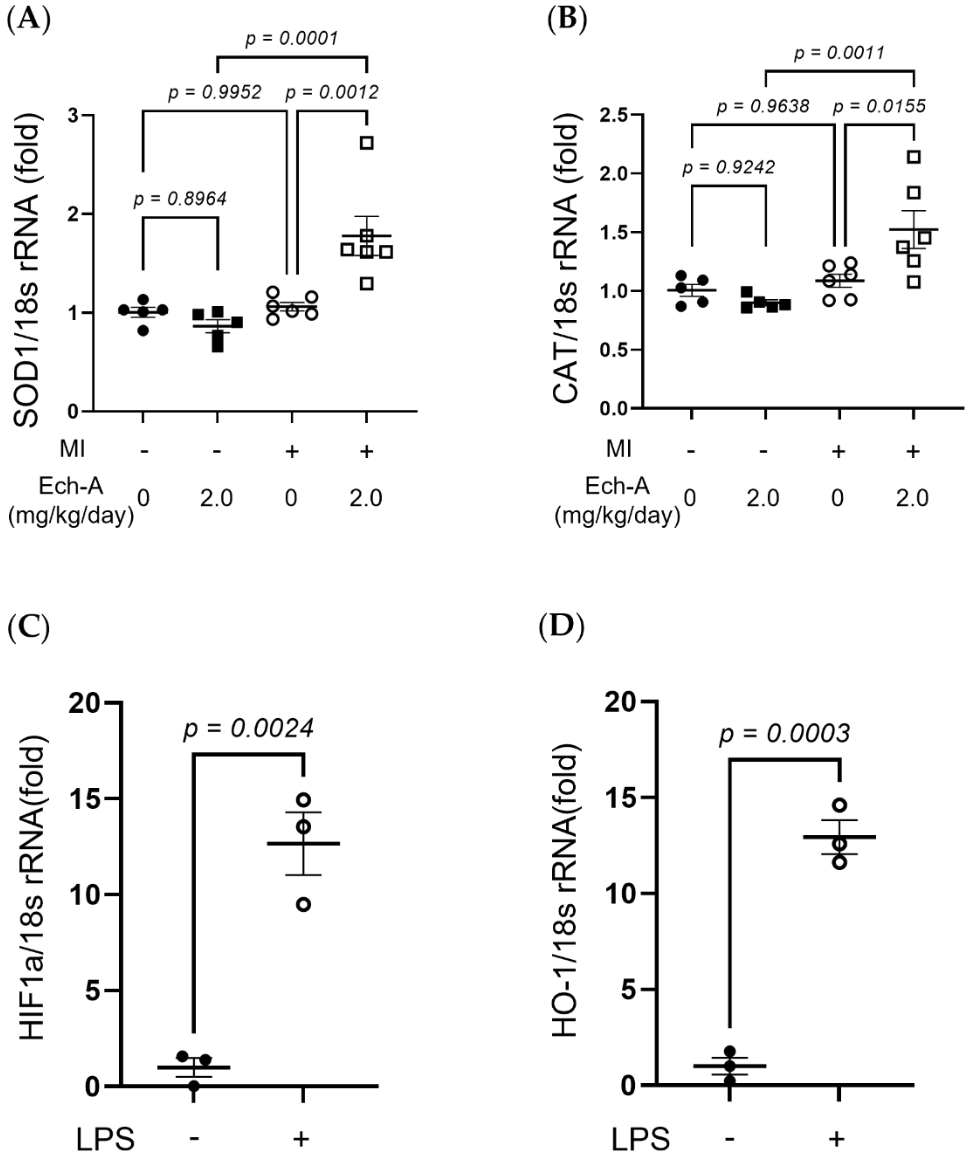

Figure S2. Gene expression levels of antioxidant enzymes in mouse heart and hypoxic markers in BMDM. (**A,B**) Expression levels of *superoxide dismutase 1 (*SOD1*)* and *catalase (*CAT*)* mRNAs in sham and MI mouse hearts 5 weeks after operation. (Sham + vehicle (●): n=5, Sham + Ech-A 2.0 mg/kg/day (■): n=5, MI + vehicle (○): n=6, MI + Ech-A 2.0 mg/kg/day (□): n=6) (**C,D**) Expression levels of *hypoxia inducible factor 1 subunit alpha (*HIF1a*)* and *heme oxygenase-1 (*HO-1*)* mRNAs in BMDM treat with or without LPS (5 ng/ml) and IFNγ (10 ng/ml) for 24 h. (Control + vehicle (●), LPS + vehicle (○): n=3) Data were presented as mean ± s.e.m. P-values were calculated using one-way ANOVA followed by Šídák's multiple comparisons test (**A,B**) or unpaired t test (**C,D**).

Table S1. Primer sequences for qPCR

**Gene**	**Primer sequence**	
18s rRNA (rodent)	Forward	ATTAATCAAGAACGAAAGTCGCAGGT
	Reverse	TTTAAGTTTCAGCTTTGCAACCATACT
SOD1 (mouse)	Forward	GGAAGCATGGCGATGAAA
	Reverse	AAATGAGGTCCTGCACTGGTA
CAT (mouse)	Forward	AAGGTTTGGCCTCACAAGGA
	Reverse	GCGGTAGGGACAGTTCACAG
HIF1a (mouse)	Forward	ACCTTCATCGGAAACTCCAAAG
	Reverse	CTGTTAGGCTGGGAAAAGTTAGG
HO-1 (mouse)	Forward	AAGCCGAGAATGCTGAGTTCA
	Reverse	GCCGTGTAGATATGGTACAAGGA
